# Digital consultation in primary healthcare: the effects on access, efficiency and patient safety based on provider experience; a qualitative study

**DOI:** 10.1080/02813432.2022.2159200

**Published:** 2022-12-26

**Authors:** Pär Eriksson, Tora Hammar, Stefan Lagrosen, Evalill Nilsson

**Affiliations:** Department of Medicine and Optometry, Linnaeus University, Kalmar, Sweden

**Keywords:** Primary healthcare, e-health, telemedicine, provider experience, digital technology

## Abstract

**Objective:**

The objective of the study was to explore the experiences of healthcare staff working with and being part of the implementation of a digital platform for patient-provider consultation across quality dimensions of access, efficiency, and patient safety.

**Design:**

The study uses qualitative design to investigate experiences and the views of healthcare professionals. Data collection combined semi-structured individual and focus-group interviews. Content analysis was used to identify categories within the content areas ‘access’, ‘efficiency’, and ‘patient safety’.

**Setting:**

The basis for the study was an e-consultation platform introduced in three primary healthcare centres in the County of Kalmar in southeast Sweden in 2019.

**Results:**

Healthcare staff experienced that the platform offered an open channel for communication with patients in need of frequent contact. This reduced anxiety and therefore the frequency of follow-up appointments. Healthcare staff also noted that the platform offered flexibility in contact benefitting patients with mental health problems. These patients were found to make contact through the platform after closing hours when problems were more acute or intense. However, the risk of digitally illiterate groups being excluded was also noted. Efficiency gains were identified among patients with simple cases which were handled more quickly through the platform. However, low uptake and the experience that the platform did not replace, rather was added on top of other already existing functions and procedures, negatively affected the overall efficiency. Standardized questions in automated medical history-taking contributed to patient safety.

**Conclusion:**

The findings suggest that text-based e-consultation platforms may bring important quality improvements to primary healthcare service in terms of access, efficiency, and patient safety. Yet, areas where e-consultation does not contribute to quality improvements puts important quality gains at risk.

**KEY POINTS**
- Text based digital consultation improved access for patients in need of frequent appointments and for patients with mental health problems.
- Efficiency gains among patients with simple cases, and in dealing with patients with mental health problems were noted. However, lack of confidence in platform functions due to low uptake, and limited control over work situation, were perceived as negatively affecting overall efficiency.
- Health care staff experienced improved patient safety through a standardized set of questions in automated medical history-taking.

## Introduction

Development in primary healthcare in Sweden today is characterized by a strong focus on digital transformation and its contribution to improvements in service delivery [1–3]. This is in-line with key policy documents advocating for the adoption of digital innovations in primary healthcare with the aim of reducing practice workload and responding to expectations from the patients [4–7]. However, the mandate of primary healthcare is complex. It serves as the first point of contact and gatekeeper of more specialised care. It provides for comprehensive care needs over time - acute, preventive as well as chronic. Developments in demography and epidemiology add to changes in demand for services. For example, an increasing number of older people in the population creates a growing need for continuity of care, often homebased. Likewise, the COVID-19 pandemic highlighted the need for options for remote access to care to reduce the risk of spread of infection.

Similar to other countries, digital platforms for provider-patient communication have gradually been introduced into the Swedish primary healthcare system during the past decade [8–10]. Studies report varying results; recurrent themes include increased access to services for those who use them [11], especially when combined with continuity of care [12]. Other studies report digital consultation primarily attracts the well-off, young people, and parents seeking care for their children [2]. Studies looking at physicians’ job control, demand, and support, conclude that medical skills and abilities are at risk if service is solely based on e-consultation, suggesting integration with traditional office-based services to add value [13–15]. Implementation of these platforms requires new routines to be set up. Evidence indicate an increasing risk of disruption of workflow [16], and that new routines take up more of healthcare professionals’ time rather than less, alternatively, that workload is simply shifted to other staff members, and potential efficiency gains are lost [17]. Finally, general practitioners using chat-based provider–patient communication found patients’ self-described medical history to benefit patient safety [18]. A more comprehensive assessment could ensure further diffusion does not lead to unnecessary costs and resistance from healthcare staff, jeopardizing critical organizational reforms in primary healthcare services.

The World Health Organization (WHO) refers to six dimensions of quality of healthcare services; effectiveness, efficiency, access, patient centeredness, equity, and safety [19]. These dimensions provide a useful framework for assessment of e-health innovations bringing quality aspects to the forefront in a comprehensive way, with the intention to advance quality in day-to-day services.

The focus of this study is one of the chat-based patient-provider communication platforms currently available on the Swedish market (referred to as ‘the platform’ in this paper). It was implemented in three primary healthcare centres (PHCC) in Region of Kalmar County in Sweden in 2019. The purpose of this study was to explore the experiences of healthcare staff working with and being part of the implementation of the platform across quality dimensions. As the study was focusing on staff experiences and did not include neither patient experiences, not quantitative data, the dimensions of access, efficiency, and patient safety were deemed the most relevant to include.

## Methods

This study uses qualitative design to understand the experiences and views of health care staff working with and being part of the implementation of the platform. Data collection combined semi-structured individual and focus-group interviews. Qualitative content analysis was chosen as it offers a systematic methodology for testing and grouping qualitative data.

### Setting

The Swedish healthcare system is decentralized and independently organized by 21 regional health authorities. Primary care is largely provided by multi-professional PHCCs offering a range of services to registered patients. Reimbursement is based on a combination of capitation and fee-for-service. Several different e-consultation platforms are currently in use in Sweden by both public and private healthcare providers. The rapid growth of private companies in Sweden, offering direct-to-consumer digital consultation [2,11] to anyone without prior registration, has exacerbated the need for a similar scale-up of e-health consultation services in the public sector. This is primarily prompted by the fact that the cost of these appointments falls back on the PHCC where the patient is registered.

The three PHCCs where the platform was being piloted had ∼22,000 registered patients from both rural and urban communities. The Care Need Index (CNI) varied from 0.97 to 1.05. The corresponding range among all PHCCs in the County of Kalmar at the time was 0.72 to 1.56, where a CNI above 1 indicates a higher care need. The study period was 6 months starting in September 2019 and ending in February 2020 (Table 1).

**Table 1.** Number of registered patients, number/share of visits, CNI, and location during the study period.

PHCC	Number of registered patients	Number/ share of visits	CNI	Location
PHCC 1	10,399	292/2.8%	0.97	Rural
PHCC 2	3787	281/7.4%	1.03	Rural
PHCC 3	7708	400/5.2%	1.05	Urban
Total	21,894	973/4.4%		

PHCC: Primary Health Care Centre. Number of registered patients indicates the average number during the six-month study period. Number/share of visits indicates the total number of registered patients contacting the PHCC via the e-consultation platform during the study period and the share in relation to number of registered patients. The Care Need Index (CNI) is calculated by Statistics Sweden (SCB). It measures the risk of ill health in the population.

All the PHCCs offered patients several options for contact; for example, patients could book appointments or renew prescriptions by telephone or online. The PHCCs also offered drop-in consultations. The platform was advertised through the website of each PHCC, where patients would normally look for contact information. Access was also possible *via* a link that could be sent by PHCC staff directly to the patient in a text message. The medical history-taking was based on dynamic templates adaptive to search causes, generating questions throughout the process. The system compiled the medical history into a comprehensive report. Free-text options were also available. Patients were informed they would be contacted no later than 3 h after submitting a request during normal office hours. Contact was made *via* asynchronous chat unless the triage nurse deemed the case sufficiently urgent to refer the patient to the emergency department. During the chat, pictures and forms could be exchanged and additional staff could be consulted if needed. Documentation was manually added to the electronic health record for the individual patient when the case was closed. All members of staff identified as active users of the platform were trained by the supplier on how to use the functions of the platform. A support group was also formed consisting of members of the staff at the three PHCCs and technical experts from the supplier to oversee the implementation and adjust technical and organisational issues as need arose. The training and the support did not include issues related to provider-patient interaction during the chat (or video) through the platform.

### Sampling

The practice manager at each PHCC identified the group of staff actively involved in the day-to-day running of the platform. Respondents were strategically sampled from among the identified group. The aim was to reflect a balanced mix of different staff categories and ensure an even distribution of participants from the three PHCCs. In total, 19 members of staff were invited and 18 were interviewed (one was unable to attend). The group consisted of 14 women and four men. These included eight general practitioners (GPs), eight nurses, and two secretaries. Individual interviews were conducted with 14 of those recruited (five GPs, seven nurses, and two secretaries/administrators). Four members participated in a focus group interview (three GPs and one nurse) (Table 2).

**Table 2.** Staff categories by PHCC included in the study (*n* = 18).

PHCC	GP	Nurse	Secretary/admin	Total
PHCC 1	3	2		5
PHCC 2	2	4	1	7
PHCC 3	3	2	1	6
Total	8	8	2	18

PHCC: Primary Health Care Centre; GP: General Practitioner.

### Data collection

All interviews were conducted by the first author (PE) using a semi-structured interview guide. Probes were used to stimulate discussion. Individual interviews were conducted *via* phone between 20 May and 5 June 2020. The focus group discussion was carried out with the four members of the steering committee of the pilot project. The steering committee members were all linked to one of the PHCCs and involved in the day-to-day running of the platform. The purpose of combining interview techniques was to further pursue views and opinions [20], and to ensure the completeness of and confirm data [21,22]. The focus group interview was conducted using a video conferencing system. Before the interviews, information about the study was distributed to all participants and all participants gave informed consent. Individual interviews lasted between 15 and 35 min and the focus group discussion was 54 min. Audio recordings were made of all interviews, and these were transcribed verbatim. The transcript consists of 75 pages of single-spaced text.

### Data analysis

Deductive content analysis using an unconstrained matrix as described by Elo and Kyngäs was used, as it allows for the testing of categories, concepts, or models [23]. The interview transcripts were initially read by the first author to form a general understanding of the data set. Following the structure of the investigation, based on dimensions of quality the first author together with two other authors (TH and EN) developed a categorization matrix containing the content areas ‘access’, ‘efficiency’, and ‘patient safety’. Different categories were then formed by the first author within the bounds of the matrix following the principles of inductive content analysis, and relevant quotes were identified and translated into English. Meaning units were identified during a process of reading and re-reading by all authors. These were then condensed into subcategories. In the next step, preliminary main categories were formed and discussed among the research team. A second reading was performed and discussed to further pursue views and experiences expressed by the interviewees to ensure rigour at all levels of the analysis, and to finalise the set of main categories [24] (Table 3).

**Table 3.** Examples of meaning units, condensed meaning units and sub categories—content area: efficiency.

Meaning unit^a^	Condensed meaning unit	Sub category
‘…we have about 10 different inboxes … that we should monitor preferably every day… everything including lab answers, and notes, and e-mails, and messages internally, and prescriptions, and all. There are ten different places we have to keep track of, sign off for… And [the e-consultation platform] has somehow become just another such inbox, and what I and my colleagues experience is that it is quite stressful’ [*interview #08*]	[The e-consultation platform] becomes just another inbox to keep track of and it contributes to stress	Adding on responsibilities
‘You must not forget that you have to do it at certain times of the day. So, it becomes an additional thing we must do’ [*interview #01*]	[The e-consultation platform] becomes an additional responsibility	

^a^Relevant quotes translated into English.

## Results

The analysis yielded a total of 24 subcategories and 10 main categories. Four main categories belong to the content area *Access*, four to the content area *Efficiency*, and two to the content area *Patient Safety*. Each main category is described in detail below (Table 4).

**Table 4.** Subcategories, main categories, and content areas.

Sub category	Main category	Content area
Taking part in development of digital care	Digital access a necessary complement to already existing access points	Access
Offering additional access points		
Requires access to appropriate devices	Risk of exclusion	
Requires being digitally literate		
The system enables continuous access to care for certain patient groups	Enabling continuous access reduces anxiety and the need for frequent appointments	
Continuity in access contributes to reduced anxiety and therefore less need for contact with the PHCC		
The possibility of using different means of communication	Flexibility in communication improves access	
Patients initiate contact outside of office hours		
Sensitive to patients’ need		
Facilitates easy contact between patient and caregiver		
Causing double work	Lack of confidence regarding the digital tool limits efficiency in work process	Efficiency
Communication back and forth		
Process not clear		
Limited value of automated patient history taking		
Limited experience causing disruption	Lack of control over work situation reduces efficiency	Efficiency
Adding on responsibilities		
Creating opportunities for patients with mental health problems to express their needs	Efficiency gains identified for certain patient groups	
Creating assurance among patients in need of frequent contacts		
Patients using multiple providers for the same concern	Overall efficiency suffers when patients are using multiple/parallel ways of contacting healthcare services	
Patients using multiple access points to access same provider		
Standardized questions contribute to good quality and patient safety	Improved medical history-taking	Patient safety
Patients use their own words to describe their problems		
Information is not lost		
Medical history not altered when handed over	Medical history not altered when handed over	

### Access

#### Digital access is a necessary complement to already existing access points

The additional option of initiating contact with the PHCC using the platform was seen as a good complement to the already existing access points. Even though uptake was limited and access to services, in general, was considered good, respondents claimed patients expected a digital option. Staff feared patients would lose confidence in the services unless these were made available in the same way people access other basic services in society.

*‘The patients are obviously asking for it and I also think it is a good complement’ [interview #09]*

#### Risk of exclusion

It was recognised that offering access to services through the platform required certain capabilities from the patients. Not having access to appropriate devices, such as a computer, tablet, or smartphone, or being digitally illiterate were identified by the respondents as potential obstacles. However, it was also noted that the platform would not necessarily impair access to services for these patients given other already existing alternatives remained intact, which was the case. Maintaining existing access points to services for those patients not conversant with digital devices was seen as a prerequisite for acceptance and a successful e-consultation service roll-out.

#### Enabling continuous access reduces anxiety and the need for frequent appointments

The platform presented an unexpected feature of communication with a positive impact on access to services. Respondents noted reduced anxiety among the patients which needed frequent appointments and follow-up appointments. The staff pointed out that the platform seemed to create a sense of continuous access for these patients. Nurses responsible for patients with chronic diseases, such as asthma/chronic obstructive pulmonary disease (COPD), or patients on long-term sick leave, described how they used the features of the platform for monitoring and follow-up and to make dose adjustments for prescribed drugs, thus reducing the need for these patients to repeatedly initiate contact with the PHCC. As a result, waiting times for patients in need of contacting the PHCC by telephone were noticeably shorter. This way of using the platform was not part of the training or instruction during implementation.

*‘I use it a lot in my work as a rehab coordinator, having an open contact with my patients creates a sense of security among them and they don’t have to make a phone call every time they have a query.’* [interview #05]

#### Flexibility in communication improves access

Respondents appreciated the fact that the platform offered flexibility in communication with patients. Communication on the platform could be in writing (chat), photographs could be uploaded and questionnaires could be shared for the patients to complete and return. Respondents noted that patients suffering from mental health problems, such as anxiety and depression, also benefitted from the flexibility in communication offered by the platform. Respondents described how it appeared to be easier for these patients to write about their problems as opposed to formulating their concerns orally. The fact that contact could be initiated outside of office hours, at weekends, or late in the evening also seemed to help these patients by giving them a sense of access to services when they needed it.

*‘Patients suffering from anxiety and depression often initiate contact in the evenings or during the night when they are feeling worse, which is better than waiting until the morning when the problems have subsided…’* [interview #11]

### Efficiency

#### Lack of confidence regarding the platform limits the efficiency of work processes

Several aspects of working with the platform were associated with a lack of confidence in the platform’s functions and the limitations it places on the efficient handling of a case. For example, respondents were hesitant to accept the accuracy and reliability of the automated medical history-taking tool, and the extent to which it correctly reflected the complaints described by patients. Sometimes this was a matter of patients lacking the precise language needed to make a diagnosis. As the procedure for taking down the patient’s medical history deviated from a traditional patient interview, additional reassurances were needed for staff to understand the complaint correctly. This could result in lengthy conversations back and forth between the nurse or doctor handling the case and the patient, often culminating in a telephone call to clarify the issues, thereby causing double work rather than reducing the time needed to close the case.

*‘The most important thing we do is to somehow find out what the patient needs help with, it is the medical history. We actually ask quite a lot of questions in the course of a short time, usually during a conversation over the phone, it is difficult to do in a chatroom, it is not as effective.’* [interview #04]

Respondents also expressed frustration about work processes related to the platform not being clear and agreed upon among the staff, and the effect this had on efficiency; for example, regarding whether or not a case should be considered closed, and procedures once a case was closed. It was reported that cases could unintentionally be left unattended because it was unclear who was responsible. This caused frustration among staff, leaving them with the impression that cases generated by the platform generally took longer to conclude than other cases.

#### Lack of control over work situation reduces efficiency

Although the e-consultation platform had been in place for a while, staff felt that they had limited experience of working with it. The anticipated high inflow of patients using the platform did not materialize. Working on the platform, therefore, became the exception rather than the rule, causing uncertainty and stress that cases were perhaps not being attended to. The platform presented new and unexpected demands while existing procedures remained in place. Contrary to expectations, it added to the multitude of day-to-day responsibilities, increasing an already high stress level at the PHCC, and therefore reducing overall efficiency.

*‘… we have about 10 different inboxes … that we should monitor preferably every day… everything including lab answers, and notes, and e-mails, and messages internally, and prescriptions, and all. There are ten different places we have to keep track of, sign off for… And* [the platform] *has somehow become just another such inbox, and what I and my colleagues experience is that it is quite stressful’* [interview #08]

#### Efficiency gains identified for certain patient groups

Nevertheless, important efficiency gains were identified for certain patient groups and certain cases. Simple cases, for example, could quickly be resolved by sharing photographs and holding asynchronous chats instead of having to find time for a telephone call or exchanging messages on voicemail. Respondents also noted that a contact initiated *via* the platform by patients suffering from anxiety or depression generated more useful information than a traditional appointment might be expected to do. The platform offered a combination of written information submitted by the patient and easy sharing of questionnaires and rating scales by the staff. With this combined information, the doctor could take clinical decisions more quickly, thus reducing the time spent on the case by both the patient and staff.

*‘… patients with anxiety who cannot express their problems during a physical meeting when they sit here because all energy is used up to keep a straight face, and then for other reasons it becomes a* [platform] *case, and suddenly I get a lot of useful information, information I have not received during two previous face-to-face consultations. The* [platform] *gave me much, much more information about how in fact the patient was feeling. Then I had enough for a referral to the psychiatric outpatient clinic.’[FGI; R1]*

#### Overall efficiency suffers when patients are using multiple/parallel ways of contacting healthcare services

Digital access to the healthcare services offered by the platform was only one of several options for initiating contact available to patients registered at any of the PHCCs. It was noted by the respondents that some patients initiated contact using several access points (i.e. by phone and on the platform) simultaneously for the same complaint. Similarly, some patients initiated parallel contacts, again for the same complaint, with both the PHCC where they were registered as a patient, and any of the national private healthcare companies offering digital consultation. This resulted in additional work, confusion, and frustration, as the same case could be handled by several different health care professionals each unaware of the others, or alternatively, the case had already been resolved by another (national) provider without notification to the PHCC where the patient was registered.

*‘I handle a case [via the platform] and then I see the same patient has called in and talked with my colleague’* [interview #13]

### Patient safety

#### Improved medical history taking

The fact that automated medical history-taking included a set of standardized questions was perceived by respondents as contributing to patient safety. Having a standardized set of questions ensured that all relevant aspects related to the patient’s situation were covered and nothing was omitted. The fact that the patients had the opportunity to describe their complaints in their own words was also considered an improvement in patient safety. As such, the medical history was not edited or interpreted. Respondents underlined the positive effect of this in terms of avoiding prejudice against the patient on the part of health care staff.

#### Medical history not altered when handed over

Respondents also highlighted that the integrity of the information produced by the automated medical history function was not compromised. Information about the patient was not altered when transferred between staff within the PHCC.

## Discussion

Using quality dimensions to understand how e-health innovations impact on the provision of healthcare services provides a useful framework for advancing quality in the day to day practice. The findings from our study highlight areas where the platform impact on quality of services, increasing the understanding of how it could be of best use. Recognising the importance of the implementation process, which has been addressed in several other studies of e-health innovations including similar platforms, and the time and effort it takes to put in place new routines in already existing everyday work practices [25], our study also brings to the forefront areas where quality gains could potentially be made and where further development of similar platforms should direct its focus.

### Quality dimension: access

Platforms for provider-patient communication have been found to increase the risk of exclusion of elderly, and patients not being digitally literate or not in possession of proper devices [12,16], These effects are considered making contact through similar platforms exclusive to the young and the well-educated, excluding elderly or vulnerable patients [26]. To avoid a similar development and given the broad and comprehensive mandate of primary healthcare the results from our study suggest that digital access through patient-provider communication platforms should be offered as a complement to already existing access points and not replace them.

The platform offered several benefits in terms of improving access to services, for example enabling an open channel for communication with patients in need of frequent contact, reducing their anxiety among them and thus the need for frequent appointments and follow-up appointments. Other studies have reported similar benefits, especially in combination with continuity of care [12], suggesting that the further development of e-consultation platforms should include applications and training in this area.

The results suggest that the platform could in fact off-load traditional points of contact and thus improve access for those patients not being capable of using digital services, such as the platform. However, further research is needed to establish the extent of these effects.

Benefits for patients with mental health problems were also noted. Staff experienced that these patients benefitted from communicating in writing rather than having a telephone conversation with a triage nurse. Other studies report similar findings from mental health nurses in primary care experiencing that embarrassment and shame can present a barrier for mental health patients to seek care, given that their concerns are often difficult to express in a conversation [27]. These findings, together with the flexibility offered through the platform to make contact when the problems were more acute or intense, suggest that services became more sensitive to the specific needs of these patients. If this is true it is an important quality improvement for this patient group. The potential risk of not picking up life-threatening conditions in time in these instances has to be acknowledged and addressed in further development of the software. A chatbot could potentially provide additional support and guidance until contact is established with health care staff at the PHCC. These findings are more prominent in our study than in other studies looking at similar e-consultation platforms [12,16,28].

### Quality dimension: efficiency

Results from our study echoes finding from other studies of similar digital platforms for patient-provider communication that efficiency gains are made in relation to handling simple cases [14,16,29], offering a combination of written communication and easy sharing of photographs, questionaries, and rating scales. For these cases, the platform was reported to save time for both health care staff and the patients [11]. Especially in relation to patients in need of frequent follow-up appointments [12]. Furthermore, Important efficiency gains were identified when dealing with patients with mental health problems. The platform provided the means for these patients to submit their queries or complaints so that the GP could take clinical decisions more quickly than might otherwise have been the case, and there was no longer any need for additional follow-up to ascertain the nature of the problem.

However, as complicated cases took more time to handle through the platform, the experience from the respondents in our study was that the overall efficiency gains were limited. Again, this could also be an effect of the limited uptake among the patients in general and the fact that both patients and staff were unaccustomed to the functions of the platform [28]. Furthermore, as the platform did not replace any of the other functions at the PHCC, but rather was considered an adjunct to already existing processes and procedures, reported benefits remained limited. This is again highlighting challenges related to the implementation and uptake of an innovation in an organisation. Frennert et al. [25] describe the process to implement and sustain a similar platform and its practices into everyday work processes and found that new routines and practices were accepted if staff perceived them as beneficial. The findings suggest that similar platforms might do well to target specific patient groups where benefits are identified rather than the general population.

### Quality dimension: patient safety

The fact that the platform provided a standard set of questions for medical history was appreciated by the staff. They linked this to the integrity of the patients and to patient safety. As the same procedure for medical history-taking applied to all patients initiating contact with the PHCCs through the platform, regardless of who they were, it was perceived by the respondents to reduce the risk of prejudice and bias against patients on the part of the staff. This is an important finding, since bias and prejudice among health care staff towards patients are known to exist and does pose a potential threat to patient safety [30,31], and could also affect equity in care. However, more research is needed to understand the extent to which automated medical history-taking contributes to improving patient safety.

### Strengths and limitations of the study

Even though the sample size was small, the three PHCCs included in the study reflect a population of varying socioeconomic status, and from both rural and urban settings. In addition, the group of respondents were strategically sampled to represent staff categories across the three PHCCs dealing with the platform. Finally, the accumulated experience of the research team provided a high standard of quality in the research process, adding to the trustworthiness of the findings. Nevertheless, the sample size, including only three PHCCs, is a limitation of this study. As the project was fixed in number and location, additional units could not be added to the sample. Also, the fact that only staff actively working with the platform were invited to participate in the study affects the credibility of the findings. The inclusion of additional staff not actively engaged in and using the platform in their day-to-day work could potentially have contributed to additional information relevant to this study. Finally, the lower-than-expected number of patients using the platform during the pilot period further limits the credibility of the findings.

## Conclusion and implications

The conclusion from this study is that digital platforms for patient-provider communication can contribute to important quality improvements in existing working methods within primary healthcare, but also that several factors have emerged that challenge both accessibility, efficiency, and patient safety, which need to be studied further. Expectations of digital innovations are high but failure to identify more precisely where e-consultation contributes to improved quality increases the risk of loss of these.

## Data Availability

Data is available upon request.
